# In Vivo Micro-Computed Tomography for Evaluation of Osteogenic Capability of Dental Pulp Stem Cells Under the Influence of Extracellular Vesicles on Alloplastic and Xenogeneic Bone Scaffolds in Rodent Intrabony Defect Model

**DOI:** 10.3390/life15121797

**Published:** 2025-11-24

**Authors:** Marius Heitzer, Philipp Winnand, Mark Ooms, Elizabeth R. Balmayor, Frank Hildebrand, Christian Apel, Zuzanna Magnuska, Fabian Kiessling, Frank Hölzle, Ali Modabber

**Affiliations:** 1 Department of Oral- and Maxillofacial Surgery, RWTH Aachen University Hospital, Pauwelsstraße 30, 52074 Aachen, Germany; 2 Department of Orthopedics, Trauma and Reconstructive Surgery, RWTH Aachen University Hospital, Pauwelsstraße 30, 52074 Aachen, Germany; 3 Experimental Orthopedics, Trauma and Reconstructive Surgery, RWTH Aachen University Hospital, Pauwelsstraße 30, 52074 Aachen, Germany; 4 Department of Biohybrid & Medical Textiles, Institute of Applied Medical Engineering, Faculty of Medicine, RWTH Aachen University, Forckenbeckstraße 55, 52074 Aachen, Germany; 5 Institute for Experimental Molecular Imaging, Faculty of Medicine, RWTH Aachen University, Forckenbeckstraße 55, 52074 Aachen, Germany

**Keywords:** stem cells, extracellular vesicles, osteogenesis, bone regeneration

## Abstract

Regeneration of jawbone defects poses major challenges. The combination of dental pulp stem cells (DPSCs) or DPSC-derived extracellular vesicles (EVs) with bone substitute materials shows promising potential for bone tissue engineering in vitro. This study evaluated the in vivo bone regeneration potential of DPSCs and EVs with bone graft substitutes in a novel intrabony defect model. DPSCs were isolated from 35 male Sprague–Dawley rat incisors, and EVs were collected from the cell culture medium. DPSCs were seeded onto alloplastic and xenogeneic bone graft materials and implanted into bone defects. Control groups received bone substitutes without DPSCs or EVs. Micro-computed tomography (µCT) was performed at 12 and 24 weeks post-implantation to assess bone volume (BV), bone density (BD), trabecular thickness (Tr.Th), bone growth rate (BGR), and bone-to-mineral ratio (BMR). Both graft types increased BV and BD, with no significant differences between them. Tr.Th increased across all treatments after 24 weeks, indicating ongoing bone remodeling. Notably, xenogeneic grafts combined with DPSCs and EVs significantly improved BGR (*p* = 0.034) and BMR (*p* = 0.021) compared to alloplastic grafts with DPSCs. Xenogeneic bone grafts combined with DPSCs and EVs appear to be a promising approach for bone regeneration of alveolar bone defects.

## 1. Introduction

Bone defects pose a great challenge to clinicians and are the result of severe atrophy, trauma, tumor resection, cyst resection [1,2], trauma, tooth loss [3], or periodontitis [4]. The most common bone defects occur as a result of tooth loss or inflammatory diseases of the periodontium, such as periodontitis, which also frequently leads to tooth loss [5]. Tooth loss typically triggers a series of biological events that alter periodontal tissue homeostasis and structural integrity mediated by local inflammation [6]. Periodontal inflammatory diseases in particular cause a loss of alveolar bone long before the loss of teeth. A common periodontitis-associated defect is the three-walled infrabony defect. In addition to successful periodontitis therapy, an important goal is the bony regeneration of bone defects to prevent the progression of jaw degeneration.

Complex cellular healing processes are the basis for complete function and regeneration without fibrous scarring of jawbone defects. In particular, the restoration of bone shape and functionality of jawbone defects is one of the major challenges of oral maxillofacial surgery [7]. Currently, there are several reconstruction options using autologous, alloplastic, and xenogenic bone grafts. Although there are considerable limitations such as limited availability, a second surgical site, and additional harvesting morbidities, the autologous bone graft is currently considered the gold standard [8,9]. Alternative augmentation techniques using alloplastic and xenogenic bone grafts have a lower osteoinductivity than autologous bone. On the other hand, these materials avoid an invasive harvesting procedure and are therefore being researched in many bone regeneration studies [3,10,11]. The use of mesenchymal stem cells (MSCs) in the form of dental pulp stem cells (DPSCs) to increase osteoinductivity has been demonstrated to have great potential for regenerative bone procedures using alloplastic and xenogenic bone substitute material [3,10,12,13].

Recent findings support the notion that therapeutic effects by MSCs are based on their paracrine secretion of extracellular vesicles (EVs), including microvesicles and exosomes [3,14,15,16,17,18]. EVs contain genetic substances such as microRNAs (miRNAs) and are an elementary component of cell proliferation and cell differentiation [19,20]. Qin et al. describe the specific and targeted modulation of miRNAs at genes of osteogenesis, whereby osteogenic differentiation of MSCs results in an induction of bone regeneration [21]. The simple extraction from body fluids or cell culture [22,23], favorable immunological interactions, and the favorable uptake of EVs by intrinsic homing effects in the target cells [24] explain the recent expansion of research on EVs [3,14,15,16,17,18].

Alloplastic, allogeneic, and xenogeneic scaffolds are among the most commonly used bone grafts, each with different biological and physicochemical properties that influence osteoconductivity and cellular response. The combination of stem cells and EVs represents a novel approach to stem cell therapy [3,14,15,16,17,18,25]. In a recent in vitro study, we were able to show that DPSCs and EVs exhibit favorable biocompatibility and osteogenic potential when they come into contact with these transplant materials. All scaffolds tested were found to support the viability, proliferation, and adhesion of DPSCs, with differences in osteogenic differentiation being material-dependent and modulated by EVs [3,10]. These findings highlight the potential of combining graft materials with stem cell-based approaches to improve bone regeneration outcomes and provide a rational basis for translational studies in rodent models.

Few in vivo studies have investigated the regenerative effects of either DPCSs or EVs [12,18,26]. In addition, these examinations were exclusively carried out in critical-size defects of the mandible with an extraoral approach outside of the oral cavity [12,18,26]. Since these models cannot adequately represent the oral situation and the mechanical effects on bone metabolism by adjacent teeth, we were able to show in a recently published pilot study in a novel rodent model with intrabony maxillary defects (dimensions: W × L × D; 1 × 1 × 1 mm) for investigation on bone regeneration that bone defects showed faster bone regeneration with the use of alloplastic and xenogeneic bone grafts compared to a control group [27].

Therefore, the aim of this study was to evaluate DPSCs and EVs using alloplastic and xenogeneic bone grafts on the bony regeneration of intrabony defects within a novel animal model.

## 2. Materials and Methods

### 2.1. The Animals

The study was conducted in accordance with ARRIVE guidelines and the German animal protection law and the EU directive 2010/63. Approval of the animal protocol was given from the Governmental Animal Care and Use Committee of the State of North Rhine–Westphalia (Approval code: AZ 81-02.04.2020.A458; Approval date: 17 August 2021). This study was performed according to Directive 2010/63 EU of the European Parliament and Council Directives on the protection of animals used for scientific purposes. In total, 35 adult and healthy male Sprague–Dawley rats were purchased in this study, with 250–300 g of body weight (Janvier Labs, Le Genest-Saint-Isle, France). Animal husbandry and all operations and examinations took place in designated areas provided by the University Hospital Aachen’s animal research department. All of the animals were housed in a pathogen-free environment in filter-top cages (Type 2000, Tecniplast, Buguggiate, Italy) with three rats per cage under a 12 h light/12 h dark cycle. The rats were provided with food (pellets) and water ad libitum, with soft-soaked food administered. Low-dust wood granulate was used as bedding (Rettenmeier Holding AG, Wilburgstetten, Germany) and as the cage enrichment nesting material (Nestlet, 14010, Plexx B.V., Elst, The Netherlands). Before the start of the experiment, the animals were kept in quarantine and housed in cages for seven days for acclimatization. The exclusion criterion before the study was a missing maxillary anterior tooth or a mucosal injury on the maxillary first molar. Humane end points were controlled daily and are defined as a 20% decrease in body weight compared to the initial weight, cramps, paralysis (trunk muscles, extremities), abnormal breathing (e.g., shallow, strained breathing), pain sounds when grabbing, auto-aggression, lack of escape behavior and pronounced soft tissue/bone infections. Pulse rate and temperature are considered secondary parameters and are taken into account if there are indications of stress. Temperature and pulse rate are measured in comparison with healthy animals using a thermometer or pulse cuff.

### 2.2. DPSC Extraction

**Figure 1:**
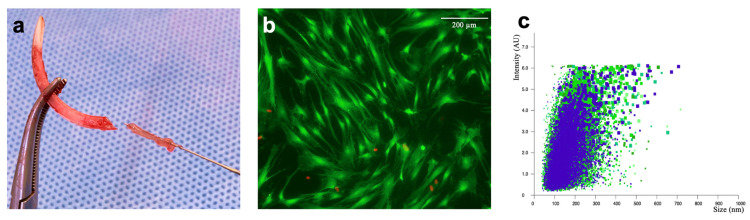
(**a**) The pulp tissue was carefully separated from the removed lower incisors by the use of sterilized endodontic files. (**b**) Image of fluorescence Live/Dead Cell Assay Kit (PromoCell GmbH, Heidelberg, Germany) of DPSC after 7 days. Green represents living cells, and red represents cells with damaged cell membranes. 50× magnification. (**c**) Nanoparticle tracking analysis (NTA) of EV with NanoSight NS300 (Malvern Panalytical, Worcestershire, UK).

For the extraction of the lower incisors and the obtaining of stem cells from the dental pulp, general anesthesia was administered by subcutaneous injection of buprenorphine (0.05 mg/kg body weight) and intraperitoneal injection of sodium pentobarbital (30–50 mg/kg body weight). A total of 35 pulps from extracted lower incisors of 35 rats were used to obtain DPSCs according to a protocol previously described [10]. After dental extraction, the pulp tissue was carefully separated from the crown and root using sterilized endodontic files (Figure 1a). Prior to isolating DPSC, a mixed enzymatic solution was prepared. This solution included 1 mL collagenase type I (12 mg/mL, cat. no. 2357206, Gibco, Grand Island, NY, USA) and 1 mL dispase solution (16 mg/mL, cat. no. 2154769, Gibco, USA). Both were added to 2 mL sterile phosphate-buffered saline (PBS) containing 100 mg/mL penicillin and 100 mg/mL streptomycin. The pulp tissues were minced and transferred into the enzyme solution for digestion at 37 °C for 40 min. During digestion, vortexing was performed every 20 min to facilitate tissue breakdown. Large-cell aggregates were subsequently removed, and single-cell suspensions were obtained by passing the cells through a 70 μM cell strainer. Digestion was terminated by adding 3 mL of basic minimum essential medium (MEM, cat. no. 12571063, Gibco, USA) containing 10% (*v*/*v*) fetal bovine serum (FBS, Gibco, USA). The single-cell suspensions were then centrifuged at 360× *g* for 10 min at 4 °C. After centrifugation, the supernatant was removed, and the pellets were resuspended in a 25 cm two-cell culture flask containing 2.5 mL cell medium. The suspension was incubated at 37 °C in 5% CO_2_. The isolated DPSC were cultured in basic MEM medium (growth medium) containing 10% (*v*/*v*) FBS and 1% (*v*/*v*) antibiotic–antimycotic solution. The cultures were maintained at 37 °C in 5% CO_2_, with the medium refreshed every two days. DPSC at passages 3 to 5 were used in subsequent experiments. For osteogenic induction, the cell growth medium was replaced with osteogenic differentiation medium. This medium was supplemented with 0.1 mM ascorbic acid, 100 nM dexamethasone, and 10 mM β-glycerophosphate when cell confluence reached 80–90% (Figure 1b). For reasons of cell toxicity and optimal stem cell growth, a concentration of 0.5 mg/mL of bone substitute material was used in the cell culture fluid for the cultivation of DPSCs on the bone substitute material.

### 2.3. DPSC-Derived EV Isolation and Examination

The extraction and detection of DPSC-derived EVs were based on a previously published protocol from a preliminary study [3]: After reaching roughly 80% confluence, DPSCs were rinsed twice with PBS and cultivated for another 24 h in serum-free medium. To remove cellular debris and any unattached cells, the supernatants were collected, differentially centrifuged (300× *g*, 2.000× *g*, and 5.000× *g*) for 15 min at 4 °C, and filtered with a 0.22 μm filter; the sediment was discarded. The supernatant was then transferred to an ultracentrifuge tube and subjected twice to ultracentrifugation at 20,000× *g* for 90 min at 4 °C to pellet the DPSC-derived EVs. The pellets were re-suspended in 100 μL of sterile PBS to obtain homogeneous EV suspension and then stored at −80 °C for further application. Nanoparticle tracking analysis (NTA) was then performed to determine the quantitative concentration and particle size distribution of the DPSC-derived EVs. All samples were diluted to a final volume of 1 mL using PBS. A pre-test was performed to evaluate the ideal measurement concentration of 20–100 particles per frame; settings were implemented according to the manufacturer’s software manual (NanoSight NS300) (Figure 1c).

### 2.4. Reimplantation of DPSCs and EVs

**Figure 2:**
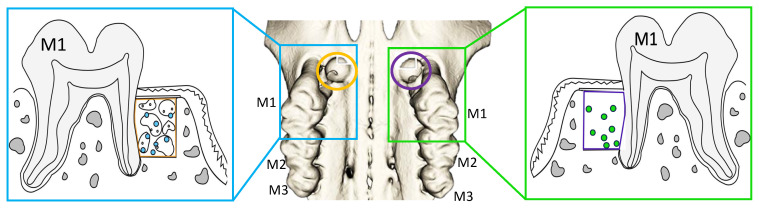
Illustration of the split-mouth model. A reconstructed rat maxilla from µCT images is shown in the center. Three-walled bone defects were created between the anterior-vestibular and anterior-palatinal roots of the first upper molar (M1). For augmentation, the bone substitute material (AP or XG) was inserted into the defects either alone (control), or combined with DPSC, or with DPSC + EV.

Operations were performed according to a previously published protocol using an operation microscope (OPMI pico f170, Carl Zeiss AG, Oberkochen, Germany) under general anesthesia by subcutaneous injection of buprenorphine (0.05 mg/kg body weight) and intraperitoneal injection of sodium pentobarbital (30–50 mg/kg body weight) [27]. Additionally, local anesthesia using articaine hydrochloride 4% was injected palatal into the first upper molars (M1) mucosa. After marginal and additionally mesial incision, the development of a mucoperiosteal flap was conducted by blunt palatal dissection of M1. Between the mesial and the first palatal root of M1, a defect with a length, width, and depth of 1 mm each was created using piezosurgery while strictly protecting the roots. Analogously, this procedure was performed on the opposite M1 in a split mouth manner. Six of the artificially created, three-walled bone defects were augmented with alloplastic bone grafts (BoneCeramic^®^, Straumann AG, Basel, Switzerland) and served as controls. Fifteen of the defects were treated using alloplastic bone substitute material with DPSCs, and an additional fourteen defects were filled with DPSCs cultivated on particulate alloplastic bone substitute material in addition to EVs. In accordance with the described procedure with the alloplastic bone graft material, six three-walled defects were treated with xenogeneic bone substitute material (BioOss^®^, Geistlich AG, Wolhusen, Switzerland) and served as controls. Fourteen defects were treated with xenogeneic bone substitute and DPSCs. Xenogeneic bone with DPSCs and EVs were inserted into fifteen bone defects (Table 1 and Figure 2). The distribution of the treatments was carried out according to a split mouth model. After augmentation, a collagen membrane (Bio-Gide^®^, Geistlich AG, Switzerland) was placed on top of the particulate grafts, and the mucoperiosteal flap was returned to its original position. Tension-free suturing was conducted with single 7-0 button sutures (Vicryl 7-0, Ethicon Inc., Raritan, NJ, USA) at the mesial incision. In total, two animals died under general anesthesia due to respiratory failure. The animals were autopsied, and no cause other than respiratory failure due to drug-induced respiratory depression could be found. Postoperative controls and inspections of the oral cavity were carried out in accordance with the approved regulations of the animal protocol. At the end of the experiment, the animals were euthanized.

### 2.5. Micro-Computed Tomography (µCT) Analysis

The micro-computed tomography (µCT) system (U-CT OI, MILabs, Utrecht, The Netherlands) was used for in vivo imaging under general anesthesia. The rats were imaged at three time points: immediately post operative (T1), after 12 weeks (T2), and after 24 weeks (T3). Imaging at T1 was performed during the ongoing general injection of anesthesia after surgery. At T2 and T3, µCT imaging was performed under general narcosis using isoflurane (induction with 5 vol% isoflurane  +  5 L O_2_/min; maintenance with 2 vol% isoflurane + 2 L O_2_/min) (Abbott GmbH & Co. KG, Wiesbaden, Germany). The µCT imaging was performed with a voltage of 65 kV, a power of 0.13 mA, and an exposure time of 300 ms. µCT scans were acquired via ultra-focus magnification through 360° rotation at 0.75° increments with 0.3 s/degree, and the data were reconstructed at an isotropic voxel size of 40 µm. Evaluation of bone regeneration was proceeded by Imalytics Preclinical software Version 3.1.3 (Gremse-IT GmbH, Aachen, Germany) according to an established protocol [28].

The region of interest (ROI) at the operation side of the upper jaw was selected and cut out of the original µCT. This kind of analysis allows the maintenance of the originally acquired high resolution and generates easier processing of in vivo scans. A fixed radius of 0.75 mm of the spherical ROI was set and placed over the artificial bone defect. For each subject and for all time points, bone tissue and implanted material within the ROIs were semi-automatically segmented using a standardized threshold of 1600 Hounsfield Units (HU). Afterwards, the segmented ROIs were analyzed in terms of changes in dimension of the three-walled defect. This operation was reproduced for each subject and for all time points. Group affiliation was not known during the analysis. Next, the segmented ROIs were analyzed in terms of bone volume (BV) in mm^3^, bone density (BD) in HU, trabecular thickness (Tr. Th) in cm, bone growth rate (BGR) in mm^3^/week, and bone-to-mineral ratio (BMR) in arbitrary units (AU). The extracted parameters were statistically compared among all the implanted materials and between different time points.

### 2.6. Power Analysis

An estimated study sample of 70 bone defects, evaluated within a split-mouth model in 35 rats with three measurements over time, was determined using the G*Power 3.1.9.6 software (Universität Düsseldorf, Düsseldorf, Germany). This sample size was calculated to achieve a statistical power of greater than 80%, with an effect size of 0.5 and a significance level (α) of 0.05, to detect differences in bone remodeling over time. The absence of relevant preliminary data or findings from similarly designed clinical studies involving DPSC and EV, as well as the lack of established evidence on the combination of DPSC, EV, and the alloplastic and xenogeneic bone graft materials, posed a challenge in conducting a power analysis. However, based on the highly significant effects observed in our in vitro investigations of bone remodeling using DPSC and EV in combination with alloplastic and xenogeneic bone graft materials [3,10], a medium effect size according to Cohen’s d was selected to detect significant changes in bone remodeling.

### 2.7. Statistical Analysis

The randomization of treatments was carried out using the Clinical Trial Randomization Tool (National Institute of Health, Bethesda, MD, USA). Initially, a simple randomization was performed to assign a bone substitute material (alloplastic or xenogeneic) to one side of the maxilla. In the next step, the treatment of the bone defect using alloplastic or xenogeneic material was software-based and assigned to the control treatment, the treatment with DPSC, or the treatment with DPSC + EV. The person performing the surgery was the only person who was informed about the group allocation. The evaluations and examinations were carried out by other persons in a blinded manner. Data were analyzed using the GraphPad Prism 7.0 program (GraphPad Software, Inc., San Diego, CA, USA). The data in this manuscript were tested for normal distribution using the D`Agostino & Pearson test and the Shapiro–Wilk test. The data were not parametric and were analyzed using the Kruskal–Wallis test. When significant effects were detected, Dunn’s multiple comparison post hoc test was applied. To control for multiple testing, the False Discovery Rate (FDR) was adjusted using the Benjamini–Hochberg procedure. All results are reported as adjusted *p*-values. All data in this paper are represented by median ± interquartile range (IQR). Differences between the groups were considered significant when *p* ≤ 0.05.

## 3. Results

**Figure 3:**
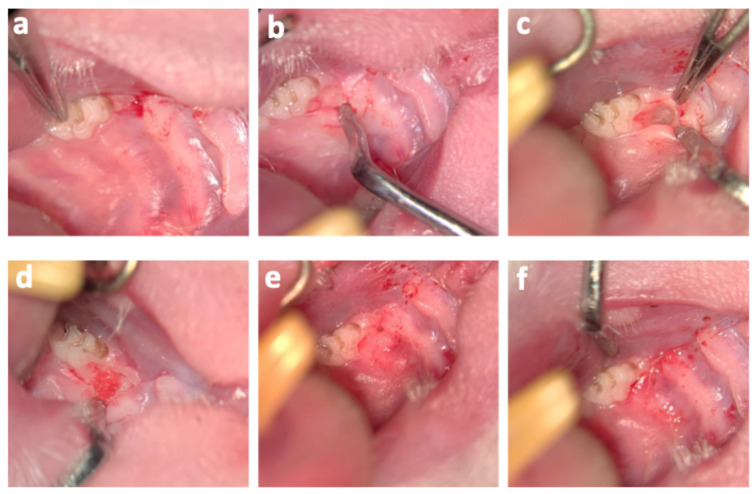
Photos of the surgical procedure: (**a**) Illustration of the incision. (**b**) Blunt subperiosteal dissection and elevation of a mucoperiosteal flap. (**c**) The surgically created intrabony defect. (**d**) Placement of particulate bone substitute material into the defect. (**e**) Tension-free adaptation of the wound margins. (**f**) Postoperative situation showing the gingival sutures.

This study investigated the osteoinductive potential of DPSC and DPSC + EV in a three-walled maxillary bone defect model in rats, focusing on the effects of two bone substitute materials. The use of alloplastic and xenogeneic bone substitute materials without mesenchymal stem cells or EVs served as a control. There were no significant differences in BV, BD, Tr.Th or BMR of absolute values between the alloplastic and xenogeneic treatments at any time during the studies, neither in the control treatment nor when DPSCs or DPSCs and EVs were used. The analysis of the relative values of BV, BD, Tr.Th or BMR showed no statistical difference. Consequently, Figure 3 exclusively presents treatment comparisons within the same bone substitute material and between treatments with DPSC or DPSCs and EVs. BV and BD were consistently higher in groups treated with xenogeneic bone graft compared to alloplastic bone graft, with BV increasing by up to 26% (0.11 mm^3^) in xenogeneic DPSCs and BD increasing by up to 11% (290 HU) in the xenogeneic control between T1 and T2, and the xenogeneic bone substitute with the DPSC and EV group exhibited significantly enhanced BGR (*p* = 0.034) and BMR (*p* = 0.021) compared to the alloplastic and DPSC group. The µ-CT reconstructions revealed clear trends, with xenogeneic-treated defects consistently demonstrating superior BV recovery and BD gains compared to AP-treated defects. Although bone resorption occurred between T1 and T2, xenogeneic bone graft treatments showed markedly enhanced regenerative potential relative to alloplastic treatments, particularly when combined with DPSC and EV therapy.

### 3.1. Bone Volume and Density

**Figure 4:**
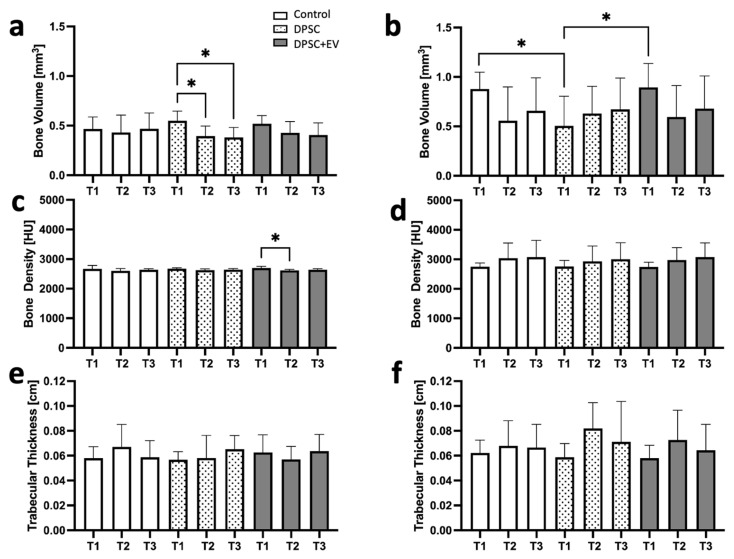
Graphical representations of the µCT analyses. (**a**) Bone volume (BV) in jaw defects with alloplastic bone substitute material. (**b**) BV in jaw defects with xenogeneic bone substitute material. (**c**) Bone density (BD) (HU = Hounsfield Units) in jaw defects with alloplastic bone substitute material. (**d**) BD in jaw defects with xenogeneic bone substitute material. (**e**) Trabecular thickness (Tr.Th) in maxillary defects with alloplastic bone substitute material. (**f**) Trabecular thickness (Tr.Th) in jaw defects with xenogeneic bone substitute material; * = *p* ≤ 0.05.

**Figure 5:**
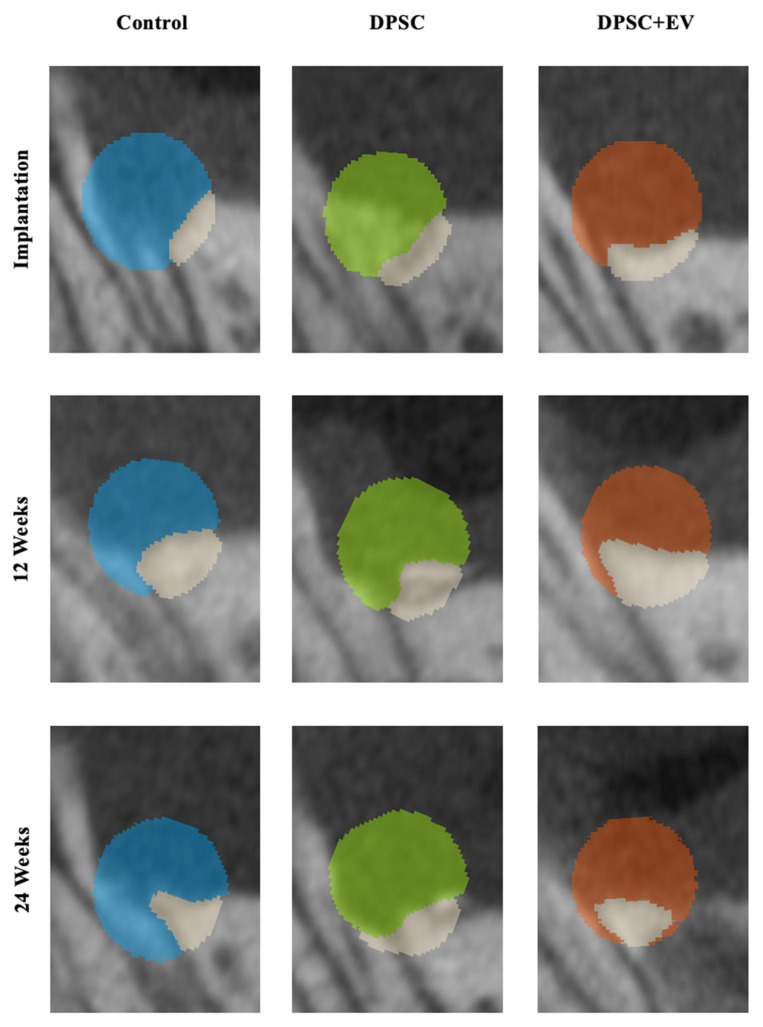
Representative two-dimensional sections from the µCT images with color coding of the ROI in the area of the periodontal jaw defects with alloplastic bone substitute material. Light blue = control; light green = DPSC; brown = DPSC + EV.

**Figure 6:**
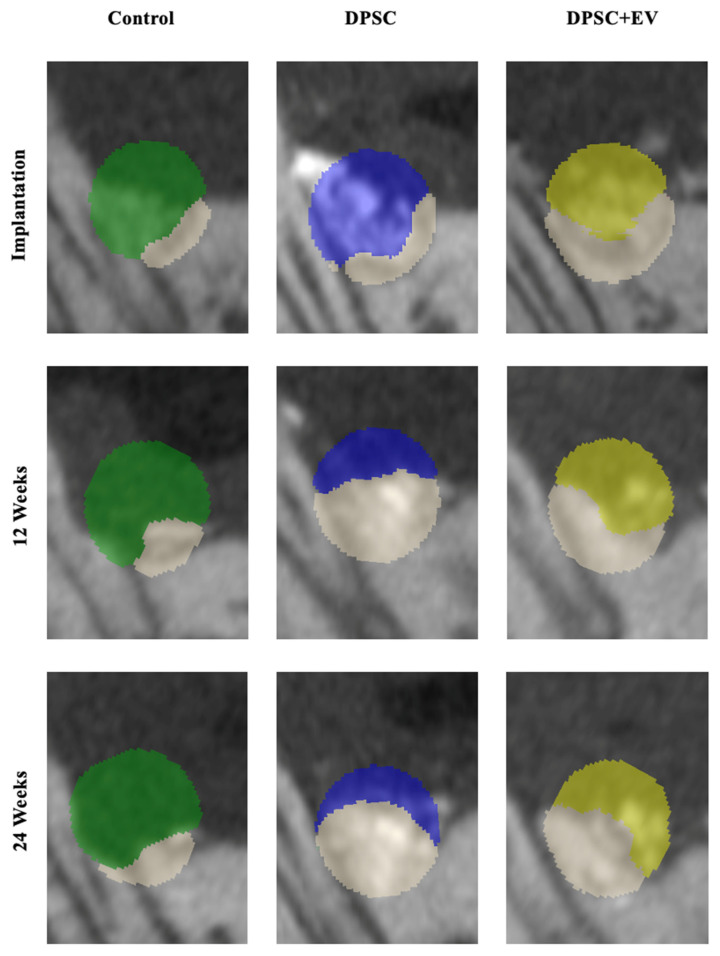
Representative two-dimensional sections from the µCT images with color coding of the ROI in the area of the periodontal jaw defects with xenogeneic bone substitute material. Green = control; blue = DPSC; yellow = DPSC + EV.

BV and BD were consistently greater in the xenogeneic bone substitute groups compared to alloplastic bone graft throughout the study. At T1, initial bone volume in the alloplastic bone graft treatment ranged from 0.42 ± 0.20 to 0.54 ± 0.12 mm^3^, which was slightly lower than in the bone defects with xenogeneic bone substitute, with 0.46 ± 0.32 to 0.92 ± 0.18 mm^3^. The relative values of the alloplastic control treatment with 0.95 ± 0.55 for T3/T1 indicate that the bone in this bone graft remained consistently (*p* > 0.009). A transient loss of BV was observed across all alloplastic bone graft application at T2, with lowest BV in the defects of alloplastic and DPSCs with 0.39 ± 0.18 mm^3^. Notably, bone defects treated with alloplastic bone graft and DPSCs showed a BV loss of 31% (−0.17 mm^3^) from T1 to T3, whereas xenogeneic bone graft with DPSC-treated defects experienced a corresponding gain of 34% (+0.17 mm^3^) over the same period (Table 2 and Table 3, Figure 4, Figure 5 and Figure 6). Moreover, BV in the xenogeneic bone defects demonstrated a recovery between T2 and T3, with an increase of up to 0.11 mm^3^, reaching a maximum of 0.61 ± 0.50 mm^3^ in xenogeneic bone graft with DPSCs and EVs at T3. BD in the xenogeneic treatments also followed an upward trend, reaching values between 2660 ± 850 HU and 3216 ± 945 HU at T3, while alloplastic-treated bone defects exhibited relatively stable BD with values between 3467 ± 265 HU and 3570 ± 752 HU throughout the study.

### 3.2. Radiological and Structural Observations

Radiological analyses showed no significant differences in Tr.Th, or BMR between alloplastic and xenogeneic bone substitute use in the absence of cellular treatments. Notably, Tr.Th increased by approximately 0.01 cm across all treatment modalities by T3, indicating active bone remodeling (Table 2). Accordingly, the values of the relative change at T2/T1 indicate an increase in trabecular bone structures in the xenogeneic bone substitute with DPSC and EV treatments, with values of 1.43 ± 0.62 and 1.14 ± 0.68, respectively (Table 3).

### 3.3. Bone Growth Rate and Bone-to-Mineral Ratio

**Figure 7:**
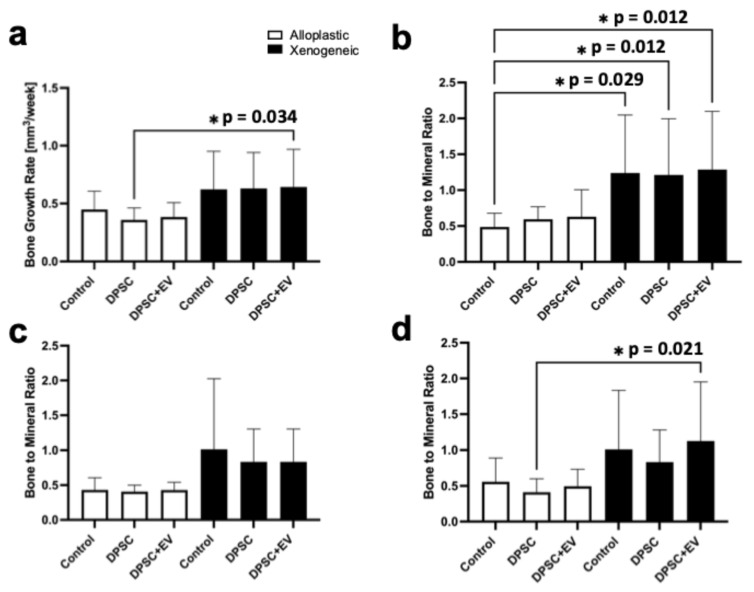
(**a**) Graphical representations of the bone growth rate. (**b**) Diagram of the bone to mineral ratio after implantation, (**c**) after 12 weeks and (**d**) after 24 weeks; * = *p* ≤ 0.05.

The combination of xenogeneic bone substitute with DPSCs and EVs demonstrated significantly improved bone regeneration metrics compared to alloplastic bone graft and DPSCs. The BGR in the xenogeneic bone substitute with DPSC and EV treatment was 0.64 ± 0.33 mm^3^/week (*p* = 0.034), significantly surpassing the rate observed with alloplastic bone graft and DPSCs, with 0.38 ± 0.12 mm^3^/week. Similarly, at T3 the BMR was significantly higher in bone defects with xenogeneic bone substitute with DPSC and EV application (0.88 ± 1.31 AU, *p* = 0.021) compared to alloplastic bone graft and DPSCs (0.48 ± 0.32 AU), reflecting enhanced mineralization and bone remodeling within the defect (Figure 7).

## 4. Discussion

The therapeutic potential of MSC and MSC-derived EVs has been extensively reported [16,21,24,25,29,30,31]. Both in vivo studies and in vitro studies have sufficiently demonstrated that many key factors of bone remodeling are controlled under the regulatory action of MSCs or MSC-derived EVs [21,25,31,32]. Especially in the field of oral and maxillofacial surgery, DPSC or DPSC-derived EVs have shown promising preliminary in vitro and in vivo results related to bone remodeling [3,10,18]. Therefore, in this study, the authors focused on the evaluation of osseous regeneration of allogenic or xenogeneic bone graft substitutes in periodontal three-wall defects under the influence of DPSCs or DPSC + EV.

In rodent studies, investigations on bone regeneration by DPSC or DPSC-derived EVs have mainly been investigated in artificial critical size defects of the bony cranium or in the area of the mandibular ramus [18,33]. Even if these studies were able to demonstrate the enormous potential of DPSC-driven therapy in pronounced bone defects [18,33], the investigations in defects of the skull or the mandibular ramus do not correspond to normal defects and therefore do not correspond to the required application for bone regeneration. The most common bony defects of the jaws include intrabody defects or burr hole defects, which occur as a result of periodontitis or tooth loss [5,34,35]. Although the bone model does not describe a defect of critical size, the major advantage of the novel jaw defect model used in this study is that it allows investigations under realistic conditions and represents the indications for bone augmentation of a three-walled jaw defect under the influence of mechanical stresses of the stomatognathic system in direct contact with the tooth surfaces.

In their recent study on bone regeneration in the jaws of rats using DPSC-derived EVs and a collagen membrane, Lee et al. [18] reported that this combination enables faster healing of the bony defect. These findings indicate that, especially, the combination of EVs derived from DPSCs and the use of a collage membrane generate supportive effects to facilitate bone defect regeneration [18]. Therefore, in this study, collagen membranes were employed as standard therapy according to socket preservation processes, and the data obtained show good bony regeneration within the three-walled maxillary defect after 12 and 24 weeks.

It has been shown that the presence of EVs accelerates regenerative processes of bone metabolism [3,14,18,36]. These favorable regenerative processes are described in particular under the combination of EVs and the presence of scaffolds [14,36]. Especially, the osteogenic differentiation of MSCs is positively associated by the presence of EVs and causes an induction of bone regeneration [21]. Analogously, in a recently published in vitro study, we were able to demonstrate that the presence of EVs can increase the osteogenic regenerative potential of DPSCs on different bone scaffolds [3]. In this study, the BGR was significantly increased in the maxillary defects with DPSC + EV and xenogenic bone material with 0.64 mm^3^/week compared to the bone defects with DPSC and alloplastic material with 0.36 mm^3^/week (*p* = 0.034). However, the positive influence of EVs on bone regeneration driven by DPSCs under the presence of bone scaffolds was generally not verified in this rodent study.

Different research approaches on the osteogenic potential of MSCs and EVs are described in the literature [3,16,21,24,25,29,30,31]. On the one hand, some studies with MSCs or EVs were conducted without the use of bone grafts [18], and on the other hand, some studies used bone grafts [3,10,36]. In recently published in vitro studies, the authors were able to show that the combination of DPSCs and xenogeneic bone graft was significantly superior to the use of alloplastic bone graft material [10]. This enormous effect of increased osteoinductivity of the in vitro findings between the alloplastic bone graft substitutes and the xenogeneic bone graft substitutes was not confirmed in our in vitro study under the complex orchestrations of bone regeneration in a living organism. The differences in the BV of the control, DPSC, and DPSC + EV treatments between the alloplastic xenogeneic bone graft substitutes and the xenogeneic bone graft substitutes tended to be increased in the bone defects with xenogeneic bone graft substitutes, and there was a general reduction in the BV in the bone defects with alloplastic bone graft substitutes over the observation period of 24 weeks. The alloplastic augmented bone defects with DPSCs showed a significant reduction in BV at T2 and T3, with a BV of 0.37 mm^3^ at T3. In the xenogeneic defects treated with DPSCs, the BV was almost twice as high at 0.61 mm^3^ at the same time point.

The resorption mechanism of alloplastic bone replacement materials completely differs from that of natural bone [37]. The material composition is the key factor that determines the rate at which the material is resorbed, which influences the resorption rate. In particular, alloplastic bone replacement materials made of hydroxyapatite/b-tricalcium phosphate are resorbed with a significant delay. Despite the proven osteoconductivity of hydroxyapatite and b-tricalcium phosphate, their resorption properties considerably differ [37,38,39]. In contrast to natural or xenogenic bone, b-tricalcium phosphate is not degraded by osteoclastic resorption but dissolves by spontaneous hydrolysis. Hydroxyapatite, on the other hand, is only slightly resorbed and can remain for up to 11 years after implantation [40]. Therefore, bone formation is stopped approximately eight weeks after transplantation of β-TCP [37]. Similarly, we observed a slight reduction in BV in the first 12 weeks after implantation of the alloplastic materials, which did not further increase during the course of the experiment. Accordingly, the BMR and the BD of the defects with alloplastic bone substitutes were almost constant with 0.40 to 0.63 AU, and 2688 to 3563 HU, respectively, for the control, DPSC and DPSC + EV treatments over the entire study period of 24 weeks in the µCT analyses.

## 5. Conclusions

Within the limitations of a rodent study, the data of the present study show that the osteogenic effect of stem cells from the pulp and its EVs in a living organism is significantly different from in vitro studies. The use of xenogeneic bone substitute material appears to have a favorable influence on bone regeneration in the treatment of three-walled periodontal bone defects in combination with DPSC, but above all with DPSC + EV against the background of the BGR and bone remodeling in the form of the BMR. The extent to which the observed regenerative effect is attributable to the xenogeneic bone substitute material or to the influence of DPSCs and EVs remains to be clarified. The use of DPSC and DPSC-derived EVs in combination with an alloplastic bone substitute material showed no additional positive impact on osseous defect regeneration. The osteogenic capability of DPSCs and EVs could be evaluated even better in larger critical-size defects. Further studies are needed to verify these findings.

## Figures and Tables

**Table 1:** Representation of the treatments of the three-walled bone defects on the upper first molar (M1) of the 35 animals.

	Alloplastic (AP)		Xenogeneic (XG)	
	Left Jaw	Right Jaw	Left Jaw	Right Jaw
Control	3	3	3	3
DPSC	8	7	7	7
DPSC + EV	7	7	7	8

**Table 2:** Analysis of in vivo µCT imaging. Parameters are indicated as absolute values (with interquartile range). Abbreviations: BV = bone volume, BD = bone density, Th.Th = trabecular thickness, BMR = bone to mineral ratio, HU = Hounsfield Units, AU = arbitrary units.

	Alloplastic (AP)									Xenogeneic (XG)								
	Control			DPSC			DPSC + EV			Control			DPSC			DPSC + EV		
	T1	T2	T3	T1	T2	T3	T1	T2	T3	T1	T2	T3	T1	T2	T3	T1	T2	T3
BV (mm^3^)	0.42 (0.20)	0.40 (0.31)	0.45 (0.25)	0.54 (0.12)	0.39 (0.18)	0.37 (0.17)	0.53 (0.06)	0.41 (0.15)	0.41 (0.21)	0.92 (0.18)	0.43 (0.33)	0.56 (0.65)	0.46 (0.32)	0.54 (0.34)	0.61 (0.50)	0.89 (0.32)	0.48 (0.26)	0.60 (0.64)
BD (HU)	2588 (648)	3400 (853)	3570 (752)	2905 (280)	3402 (441)	3549 (379)	2773 (336)	3321 (365)	3467 (265)	2738 (147)	2838 (864)	2836 (844)	2706 (440)	2601 (734)	2660 (850)	2699 (237)	2864 (834)	3216 (945)
Tr.Th (cm)	0.06 (0.01)	0.07 (0.02)	0.06 (0.02)	0.06 (0.01)	0.05 (0.01)	0.07 (0.02)	0.06 (0.01)	0.05 (0.01)	0.06 (0.01)	0.06 (0.02)	0.05 (0.03)	0.06 (0.03)	0.06 (0.02)	0.08 (0.02)	0.06 (0.04)	0.05 (0.01)	0.06 (0.03)	0.06 (0.03)
BMR (AU)	0.49 (0.22)	0.40 (0.31)	0.54 (0.43)	0.60 (0.30)	0.40 (0.14)	0.38 (0.20)	0.46 (0.47)	0.41 (0.15)	0.48 (0.32)	0.93 (1.27)	0.46 (1.67)	0.65 (1.22)	1.03 (0.79)	0.66 (0.88)	0.62 (0.69)	0.82 (1.47)	0.56 (1.68)	0.88 (1.31)

**Table 3:** Analysis of in vivo µCT imaging. Parameters are indicated as relative values (with interquartile range). Abbreviations: DPSC = dental pulp stem cells, EV = DPSC-derived extracellular vesicles, BV = bone volume, BD = bone density, Th.Th = trabecular thickness, BMR = bone to mineral ratio.

	Alloplastic						Xenogeneic					
	Control		DPSC		DPSC + EV		Control		DPSC		DPSC + EV	
	T2/T1	T3/T1	T2/T1	T3/T1	T2/T1	T3/T1	T2/T1	T3/T1	T2/T1	T3/T1	T2/T1	T3/T1
BV	0.79 (0.85)	0.95 (0.55)	0.72 (0.31)	0.68 (0.36)	0.89 (0.36)	0.87 (0.51)	0.46 (0.67)	0.62 (0.54)	0.58 (0.37)	0.63 (0.28)	0.61 (0.31)	0.66 (0.55)
BD	0.97 (0.05)	0.99 (0.05)	0.98 (0.04)	0.99 (0.04)	0.97 (0.02)	0.98 (0.02)	1.01 (0.34)	1.05 (0.126)	1.02 (0.28)	1.04 (0.17)	1.02 (0.17)	1.12 (0.18)
Tr.Th	1.33 (0.67)	1.12 (0.55)	0.89 (0.26)	1.17 (0.38)	0.89 (0.38)	0.99 (0.23)	1.02 (0.64)	1.09 (0.45)	1.43 (0.62)	1.01 (0.52)	1.14 (0.68)	1.03 (0.50)
BMR	0.97 (0.49)	1.14 (0.59)	0.68 (0.36)	0.59 (0.62)	0.86 (0.56)	0.73 (0.42)	0.64 (1.09)	0.67 (0.54)	0.69 (0.43)	0.70 (0.31)	0.89 (0.57)	0.92 (0.53)

## Data Availability

All data generated for this study are available from the corresponding authors upon reasonable request.

## Abbreviations

The following abbreviations are used in this manuscript:

DPSC	Dental pulp stem cell
EVs	Extracellular vesicles
µCT	Microcomputed tomography
Tr.Th	Trabecular thickness
BV	Bone volume
BD	Bone density
BGR	Bone growth
BMR	Bone-to-mineral ratio
